# Edge-based nonlinear diffusion for finite element approximations of convection–diffusion equations and its relation to algebraic flux-correction schemes

**DOI:** 10.1007/s00211-016-0808-z

**Published:** 2016-05-07

**Authors:** Gabriel R. Barrenechea, Erik Burman, Fotini Karakatsani

**Affiliations:** 1 Department of Mathematics and Statistics, University of Strathclyde, 26 Richmond Street, Glasgow, G1 1XH UK; 2 Department of Mathematics, University College London, Gower Street, London, WC1E 6BY UK; 3 Department of Mathematics, University of Chester, Thornton Science Park, Chester, CH2 4NU UK

**Keywords:** 65N30, 65N12

## Abstract

For the case of approximation of convection–diffusion equations using piecewise affine continuous finite elements a new edge-based nonlinear diffusion operator is proposed that makes the scheme satisfy a discrete maximum principle. The diffusion operator is shown to be Lipschitz continuous and linearity preserving. Using these properties we provide a full stability and error analysis, which, in the diffusion dominated regime, shows existence, uniqueness and optimal convergence. Then the algebraic flux correction method is recalled and we show that the present method can be interpreted as an algebraic flux correction method for a particular definition of the flux limiters. The performance of the method is illustrated on some numerical test cases in two space dimensions.

## Introduction

For an open bounded polygonal (polyhedral) domain $\Omega \subseteq \mathbb {R}^d, d=2,3$, with Lipschitz boundary, we consider in this work the steady-state convection–diffusion–reaction equation

1.1 $$\begin{aligned} \left\{ \begin{array}{lll} -\varepsilon \,\Delta u+{\varvec{b}}\cdot \nabla u+\sigma \,&{}u=f &{}\quad \text{ in } \Omega ,\\ &{}u=g &{}\quad \text{ on } \partial \Omega , \end{array} \right. \end{aligned}$$

where $\varepsilon >0$ is the diffusion coefficient, ${\varvec{b}}\in L^{\infty }(\Omega )^2$ is a solenoidal convective field, $\sigma >0$ is a real constant, and $f\in L^2(\Omega )$, $g\in H^{\frac{1}{2}}(\partial \Omega )$, are given data. In this work we adopt the standard notation for Sobolev spaces. In particular, for $D\subset {\mathbb R}^d$ we denote $(\cdot ,\cdot )_D$ the $L^2(D)$ [or $L^2(D)^d$] inner product, and by $\Vert \cdot \Vert _{l,D}$ ($|\cdot |_{l,D}$) the norm (seminorm) in $H^l(D)$ [with the usual convention that $H^0(D)=L^2(D)$].

The weak form of problem (1.1) is: Find $u\in H^1(\Omega )$ such that $u=g$ on $\partial \Omega$ and

1.2 $$\begin{aligned} a(u,v) = (f,v)_\Omega \quad \forall v\in H^1_0(\Omega ), \end{aligned}$$

where the bilinear form *a* is given by

$$\begin{aligned} a(u,v):=\varepsilon \,(\nabla u,\nabla v)_\Omega +({\varvec{b}}\cdot \nabla u,v)_\Omega +\sigma (u,v)_\Omega . \end{aligned}$$

The weak problem (1.2) has a unique solution $u\in H^1(\Omega )$ and its solution satisfies the following maximum principle (see [10]).

### **Definition 1**

(*Maximum principle*) Assume that $f\ge 0, g\ge 0$ (resp. $\le 0$) and the solution *u* of (1.2) is smooth enough. Then, if $\sigma =0$ and *u* attains a strict minimum (resp. maximum) at an interior point $\tilde{x}\in \Omega$, then *u* is constant in $\Omega$. If $\sigma > 0$, then the same conclusion remains valid if we suppose in addition that $u(\tilde{x}) < 0$ [resp. $u(\tilde{x})>0$].

This work deals with the development of a method that satisfies the discrete analogue of the last definition. The quest for such a method has been a constant for the last couple of decades. Several methods have been proposed over the years, both in the finite element and finite volume contexts (see [21] for a review). Overall, the common point of all discretisations that satisfy a discrete maximum principle (DMP) is that they add some diffusion to the equations. This extra diffusion can lead to a linear method, but it is a well-known fact that such a method will provide very diffused numerical solutions, which will converge suboptimally. Due to the previous fact, several methods that add nonlinear diffusion have been proposed.

One approach has been to add a so-called shock-capturing term to the finite element formulation. This typically amounts to a nonlinear diffusion term where the diffusion coefficient depends nonlinearly on the finite element residual, making it large in the zones where the solution is underresolved, but vanish in smooth regions. An analysis showing that nonlinear shock capturing methods may lead to a DMP was first proposed in [5], and then developed further for the Laplace operator in [6], and for the convection–diffusion equation in [7]. For a review of shock capturing methods, designed to reduce spurious oscillations, without necessarily satisfying a DMP, see [14]. More recent nonlinear discretisations, these ones based on the idea of blending in order to satisfy the DMP, are the works [1, 9], where the emphasis has been given to prove the convergence to an entropy solution. Most shock capturing techniques suffer from the strong nonlinearity introduced when the diffusion coefficient is made to depend on the finite element residual (and therefore the gradient of the approximation function). Because of this the analysis of such methods is incomplete even when linear model problems with constant coefficients are considered. In particular, in most cases uniqueness of solutions can not be proved, and the convergence theory is incomplete.

On the other hand, driven initially by the design of explicit time stepping schemes for compressible flows, so called flux corrected transport (FCT) schemes and the related algebraic flux correction (AFC) schemes were introduced [15, 19, 20]. These schemes act on the algebraic level by first modifying the system matrix so that it has suitable properties to make the system monotonous, while perturbing the method as little as possible. In the most elementary case the system matrix is simply perturbed to make it an M-matrix, resulting in a linear method. This crude strategy, however, necessarily results in a first order scheme. Then, AFC schemes introduce a nonlinear switch, or flux limiter, thus making the low order monotone scheme active only in the zones where the DMP may be violated. These schemes have also resisted mathematical analysis for a long time, but a number of results have been proved recently in [2, 3]. Indeed, in these references, existence of solutions and positivity have been proved, and a first error analysis has been performed. Nevertheless, it was shown that the DMP, and even the convergence of the discrete solution to the continuous one, depend on the geometry of the mesh.

Another approach to combine monotone (low order) finite element methods with linear diffusion and high order FEM using flux-limiters was proposed very recently in [13]. It then appears that a cross pollination between the idea of AFC and shock-capturing could be fruitful.

The objective of the present paper is to further bridge the gap between the shock capturing approach and the algebraic flux correction. Indeed we will consider a generalisation of the shock-capturing term first introduced in [4] to several dimensions, using an anisotropic diffusion operator along element edges similar to that introduced in [7]. We show that the resulting scheme satisfies the DMP and give an analysis of the method. In particular we show that the new shock capturing term is Lipschitz continuous, and, if the mesh is sufficiently regular, linearity preserving (see Sect. 2.1), which allows us to improve greatly on previous results. In Sect. 2.2 we prove existence of solutions, the discrete maximum principle, and noticeably, uniqueness in the diffusion dominated regime. We then show error estimates, which, thanks to the combined use of linearity preservation and Lipschitz continuity, turn out to be optimal in the diffusion dominated regime, for a special class of meshes (see Sect. 3). In Sect. 4, we revisit the design principles of AFC and show that the proposed shock-capturing term can be interpreted as an AFC scheme using a special flux, allowing both for a DMP and Lipschitz continuity. Some numerical results are finally shown in Sect. 5.

### Notations

We now introduce some notation that will be needed for the discrete setting. We consider a family $\{\mathscr {T}_h\}_{h>0}$ of shape-regular triangulations of $\Omega$ consisting of disjoint *d*-simplices *K*. We define $h_K:=\mathrm{diam} (K)$, and $h=\max \{ h_K:K\in \mathscr {T}_h\}$. We associate with the triangulation $\mathscr {T}_h$ the finite element spaces

1.3 $$\begin{aligned} \mathcal {V}_h:=\{\chi \in H^1({\Omega }):\chi |_K\in \mathbb {P}_1(K)\, \forall K\in \mathscr {T}_h \} , \quad \text {and} \quad \mathcal {V}_h^0:= \mathcal {V}_h \cap H_0^1(\Omega ), \end{aligned}$$

where $\mathbb {P}_\ell (D)$ is the space of polynomials of degree at most $\ell$ on *D*. The nodes of $\mathscr {T}_h$ are denoted by $\{x_i\}_{i=1}^N$, and the usual associated basis functions of $\mathcal {V}_h$ are denoted by $\{\psi _i\}_{i=1}^N$.

We let $\mathscr {E}_h$ be the set of the interior edges of $\mathscr {T}_h$. For every edge $E\in \mathscr {E}_h$, we define $h_E:=|E|$ and $\omega _E:=\{ K\in \mathscr {T}_h:K\cap E\not =\emptyset \}$, and fix one unit tangent vector, denoted by $\varvec{t}$.

For an interior node $x_i$, we define the associated edges $\mathscr {E}_i:= \{E \in \mathscr {E}_h:x_i\in E\}$ and the subset of $\mathbb {R}^d$ defined by the union of all elements *K* sharing the node $x_i$, $\Omega _i:=\{x \in \bar{\Omega }:\exists K\in \mathscr {T}_h:x \in K \text{ and } x_i\in K \}$, and the set

1.4 $$\begin{aligned} S_i:=\{ j\in \{1,\ldots ,N\}{\setminus }\{ i\}:x_j\;\text {shares an internal edge with}\; x_i\}. \end{aligned}$$

**Fig. 1:**
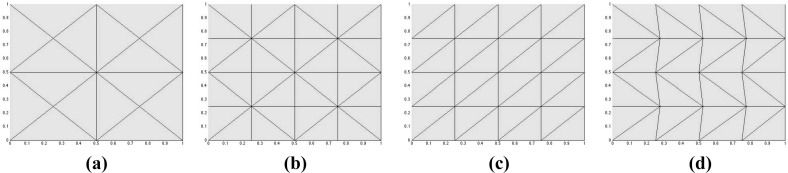
In two dimensions, meshes (**a**–**c**) are examples of symmetric meshes. Mesh (**d**) is a non-symmetric, non-Delaunay mesh

Finally, we will say that the triangulation $\mathscr {T}_h$ is *symmetric with respect to its internal nodes* if for every internal node $x_i$ the following holds: for all $j\in S_i$ there exists $k\in S_i$ such that $x_j-x_i=-(x_k-x_i)$ (see Fig. 1 for examples in two space dimensions).

## The nonlinear discretisation

The standard finite element method for the problem (1.2) takes the form: find $u_h\in \mathcal {V}_h$ such that $u_h-u_{bh} \in \mathcal {V}_h^0$ and

2.1 $$\begin{aligned} a(u_h,v_h) = (f,v_h)_\Omega \quad \forall v_h\in \mathcal {V}_h^0. \end{aligned}$$

Here, $u_{bh}\in \mathcal {V}_h$ is introduced to approximate the boundary condition *g*. Then, we propose the following stabilised method to discretise (1.2): find $u_h\in \mathcal {V}_h$ such that $u_h-u_{bh} \in \mathcal {V}_h^0$ and

2.2 $$\begin{aligned} a_h(u_h;v_h):= a(u_h,v_h)+d_h(u_h; u_h, v_h) = (f,v_h)_\Omega \quad \forall v_h\in \mathcal {V}_h^0. \end{aligned}$$

The stabilisation term $d_h(\cdot \; ; \cdot , \; \cdot )$ is defined by

2.3 $$\begin{aligned} d_h(w_h ; u_h, v_h) =\sum _{E\in \mathscr {E}_h}\, \gamma _0^{}\, h_E^d \, \alpha _E^{}(w_h)( \partial _{\varvec{t}}u_h, \partial _{\varvec{t}}v_h)_E. \end{aligned}$$

Here, $\gamma _0^{}>0$, and $\alpha _E^{}:\mathcal {V}_h\rightarrow [0,1]$ is defined as follows. First, for $w_h^{}\in \mathcal {V}_h$, we define $\xi _{w_h}$ as the unique element in $\mathcal {V}_h^0$ whose nodal values are given by

2.4 $$\begin{aligned} \xi _{w_h} (x_i) := \left\{ \begin{array}{ll} \frac{\left| \sum _{j\in S_i} w_h(x_i)-w_h(x_j)\right| }{\sum _{j\in S_i}|w_h(x_i)-w_h(x_j)|},&{}\quad \text{ if }\;\; \sum \nolimits _{j\in S_i} |w_h(x_i)-w_h(x_j)| \ne 0,\\ 0, &{}\quad \text{ otherwise }. \end{array} \right. \end{aligned}$$

Then, on each *E*, $\alpha _E^{}$ is defined by

2.5 $$\begin{aligned} \alpha _E^{}(w_h):= \max _{x\in E} \big [ \xi _{w_h}(x)\big ]^p,\quad p\in [1, +\infty ). \end{aligned}$$

The value for *p* will determine the rate of decay of the numerical diffusion with the distance to the critical points. A value closer to 1 will add more diffusion in the far field, while a larger value will make the diffusion vanish faster, but on the other hand, increasing *p* may make the nonlinear system more difficult to solve. In principle, as *p* goes to infinity the method will add the perturbations only in points with local extrema. In our calculations we have tested several different values for *p*, and have presented those for $p=1,4,8$, and 10. The higher values provide better numerical results, while keeping the nonlinear solver converging within a reasonable number of iterations. In Sect. 5 below we present a more detailed study of the behavior of the nonlinear solver with respect to the value of *p*.We finally stress the fact that, for any value of *p*, the function $\alpha _E^{}(w_h)$ is equal to 1 if $w_h$ has a local extremum in one of the end points of the edge *E*. This property is of fundamental importance for the proof of the discrete maximum principle below.

### Properties of $d_h(\cdot ;\cdot ,\cdot )$

We start noticing that

$$\begin{aligned} \sum _{j\in S_i} |w_h(x_i) - w_h(x_j)| = 0\Longrightarrow w_h|_{\Omega _i}=c\in \mathbb {R}. \end{aligned}$$

This prevents the method from adding artificial diffusion to the equations in regions in which the solution is constant. Moreover, the method is as well linearity preserving if the mesh is symmetric with respect to its interior nodes. In fact, if $E\in \mathscr {E}_h$ has endpoints $x_i$ and $x_j$, and $v_h\in \mathbb {P}_1(\omega _E)$, then

2.6 $$\begin{aligned} \sum _{l\in S_i} v_h(x_i)-v_h(x_l)=0\quad \text {and}\quad \sum _{l\in S_j} v_h(x_j)-v_h(x_l)=0, \end{aligned}$$

which gives $\alpha _E^{}(v_h)=0$. Then, the method does not add extra diffusion in smooth regions, whenever the mesh is sufficiently structured. We now state this in a more precise way. Let us decompose the stabilisation term $d_h^{}$ as the sum of edge contributions as follows:

$$\begin{aligned} d_h^{}(u_h;v_h,z_h)= & {} \sum _{E\in \mathscr {E}_h}d_E^{}(u_h;v_h,z_h)\,\\ \text{ with } d_E^{}(u_h;v_h,z_h):= & {} \gamma _0^{}\, h_E^d \, \alpha _E^{}(u_h)( \partial _{\varvec{t}}v_h, \partial _{\varvec{t}}z_h)_E. \end{aligned}$$

Then, if the mesh is symmetric with respect to its internal nodes and $E\in \mathscr {E}_h$, whenever $v_h\in \mathbb {P}_1(\omega _E^{})$, the edge diffusion vanishes, this is

$$\begin{aligned} d_E^{}(v_h;w_h,z_h)=0\quad \forall w_h,z_h\in \mathcal {V}_h^{}. \end{aligned}$$

As a consequence, if, for a given node $x_i$, with associated basis function $\psi _i$, we denote the extended macro element $\tilde{\Omega }_i:=\cup _{E \in \mathcal {E}_i} \omega _E$, then

$$\begin{aligned} d_h^{}(v_h;w_h,\psi _i)= 0,\quad \forall w_h \in \mathcal {V}_h^{} \quad \text{ and }\quad \forall v_h: v_h\vert _{ \tilde{\Omega }_i} \in P_1(\tilde{\Omega }_i). \end{aligned}$$

The next step is to show that $d_h(\cdot ;\cdot ,\cdot )$ is continuous. More precisely, it is Lipschitz continuous, and the next result is the first step towards this.

#### **Lemma 1**

For any $v_h, w_h\in \mathcal {V}_h$, and any given internal node $x_i$, the following holds

2.7 $$\begin{aligned} |\xi _{v_h}(x_i)-\xi _{w_h}(x_i)|\le 4\frac{\sum _{E\in \mathscr {E}_i}h_E|\partial _{\varvec{t}}(v_h-w_h)|}{ \sum _{E\in \mathscr {E}_i}h_E(|\partial _{\varvec{t}} v_h|+|\partial _{\varvec{t}} w_h|)}. \end{aligned}$$

#### *Proof*

It is enough to suppose that $\sum _{j\in S_i}|v_h(x_i)-v_h(x_j)|>0$ and $\sum _{j\in S_i}|w_h(x_i)-w_h(x_j)|>0$, otherwise the claim is obvious. A quick calculation gives

$$\begin{aligned}&|\xi _{v_h}(x_i)-\xi _{w_h}(x_i)|\\&\quad = \left| \frac{\left| \sum _{j\in S_i}v_h(x_i)-v_h(x_j)\right| }{\sum _{E\in \mathscr {E}_i}h_E |\partial _{\varvec{t}}v_h|} - \frac{\left| \sum _{j\in S_i}w_h(x_i)-w_h(x_j)\right| }{\sum _{E\in \mathscr {E}_i}h_E|\partial _{\varvec{t}}w_h|}\right| \\&\quad \le \left| \frac{\left| \sum _{j\in S_i}v_h(x_i)-v_h(x_j)\right| -\left| \sum _{j\in S_i} w_h(x_i)-w_h(x_j)\right| }{\sum _{E\in \mathscr {E}_i} h_E|\partial _{\varvec{t}}v_h|}\right| \\&\quad \quad + \left| \sum _{j\in S_i}w_h(x_i)-w_h(x_j)\right| \left| \frac{1}{\sum _{E\in \mathscr {E}_i}h_E|\partial _{\varvec{t}}v_h|}-\frac{1}{\sum _{E\in \mathscr {E}_i}h_E|\partial _{\varvec{t}}w_h|}\right| \\&\quad \le \frac{\sum _{E\in \mathscr {E}_i}h_E|\partial _{\varvec{t}}(v_h-w_h)|}{\sum _{E\in \mathscr {E}_i}h_E|\partial _{\varvec{t}}v_h|}\\&\qquad + \frac{\left| \sum _{j\in S_i}w_h(x_i)-w_h(x_j)\right| \,\left| \sum _{E\in \mathscr {E}_i}h_E\left( |\partial _{\varvec{t}}w_h|-|\partial _{\varvec{t}}v_h|\right) \right| }{\sum _{E\in \mathscr {E}_i}h_E|\partial _{\varvec{t}}v_h|\sum _{E\in \mathscr {E}_i}h_E|\partial _{\varvec{t}}w_h|}\\&\quad \le 2\,\frac{\sum _{E\in \mathscr {E}_i}h_E|\partial _{\varvec{t}}(v_h-w_h)|}{\sum _{E\in \mathscr {E}_i}h_E|\partial _{\varvec{t}}v_h|}. \end{aligned}$$

The following estimate can be proved in an analogous way

$$\begin{aligned} |\xi _{v_h}(x_i)-\xi _{w_h}(x_i)|\le 2\,\frac{\sum _{E\in \mathscr {E}_i}h_E|\partial _{\varvec{t}}(v_h-w_h)|}{\sum _{E\in \mathscr {E}_i}h_E|\partial _{\varvec{t}}w_h|}. \end{aligned}$$

Then,

2.8 $$\begin{aligned}&|\xi _{v_h}(x_i)-\xi _{w_h}(x_i)|\nonumber \\&\quad \le 2\min \left\{ \frac{1}{\sum _{E\in \mathscr {E}_i}h_E|\partial _{\varvec{t}}v_h|}, \frac{1}{\sum _{E\in \mathscr {E}_i}h_E|\partial _{\varvec{t}}w_h|}\right\} \, \sum _{E\in \mathscr {E}_i}h_E|\partial _{\varvec{t}}(v_h-w_h)|,\qquad \end{aligned}$$

which gives the desired result upon applying the estimate $\min \{a^{-1},b^{-1}\} \le \frac{2}{a+b}$, for two positive numbers *a* and *b*. $\square$

The Lipschitz continuity of $d_h(\cdot ;\cdot ,\cdot )$ appears then as a consequence of the previous result.

#### **Lemma 2**

The nonlinear form $d_h(\cdot ;\cdot ,\cdot )$ is Lipschitz continuous. More precisely, there exists $C_\mathrm{lip}>0$, independent of *h*, such that, for all $v_h,w_h,z_h\in \mathcal {V}_h$, the following holds

2.9 $$\begin{aligned} |d_h(v_h;v_h,z_h)-d_h(w_h;w_h,z_h)|\le C_\mathrm{lip}\gamma _0^{}h\,|v_h-w_h|_{1,\Omega }\,|z_h|_{1,\Omega }. \end{aligned}$$

#### *Proof*

We have

2.10 $$\begin{aligned}&d_h(v_h;v_h,z_h)-d_h(w_h;w_h,z_h)\nonumber \\&\quad =\sum _{E\in \mathscr {E}_h}\gamma _0^{}h_E^d\big (\alpha _E^{}(v_h) \partial _{\varvec{t}}v_h-\alpha _E^{}(w_h)\partial _{\varvec{t}}w_h, \partial _{\varvec{t}} z_h\big )_E \nonumber \\&\quad =\sum _{E\in \mathscr {E}_h}\gamma _0^{}h_E^d\alpha _E^{}(v_h)(\partial _{\varvec{t}}v_h-\partial _{\varvec{t}}w_h,\partial _{\varvec{t}}z_h)_E \nonumber \\&\qquad + \gamma _0^{}h_E^d(\alpha _E^{}(v_h)-\alpha _E^{}(w_h))(\partial _{\varvec{t}}w_h, \partial _{\varvec{t}} z_h)_E. \end{aligned}$$

The first term in the above estimate is bounded using the fact that $|\alpha _E^{}(v_h)|\le 1$, the Cauchy–Schwarz inequality, a local trace inequality, and the shape regularity of the mesh sequence, to give

2.11 $$\begin{aligned} \sum _{E\in \mathscr {E}_h}\gamma _0^{}h_E^d\alpha _E^{}(v_h)(\partial _{\varvec{t}}v_h-\partial _{\varvec{t}}w_h,\partial _{\varvec{t}}z_h)_E\le C\gamma _0^{}h\,|v_h-w_h|_{1,\Omega }|z_h|_{1,\Omega }. \end{aligned}$$

The second term is bounded next. For this, a general edge $E\in \mathscr {E}_h$ will be considered as having $x_i$ and $x_j$ as endpoints, where $x_i$ is chosen to be the vertex such that $\alpha _E(v_h)= \xi ^p_{v_h}(x_i)$. We then divide $\mathscr {E}_h=E_1\cup E_2$, where

$$\begin{aligned} E_1&:=\{ E\in \mathscr {E}_h:\alpha _E^{}(v_h) =\xi _{v_h}^p(x_i), \alpha _E^{}(w_h)=\xi _{w_h}^p(x_i)\},\\ E_2&:=\{ E\in \mathscr {E}_h:\alpha _E^{}(v_h) =\xi _{v_h}^p(x_i), \alpha _E^{}(w_h)=\xi _{w_h}^p(x_j)\}, \end{aligned}$$

and the second term in (2.10) reduces to

$$\begin{aligned}&\sum _{E\in E_1}\gamma _0^{}h_E^d\big ((\xi _{v_h}^p(x_i)-\xi _{w_h}^p(x_i))\partial _{\varvec{t}}w_h, \partial _{\varvec{t}} z_h\big )_E\\&\quad +\sum _{E\in E_2} \gamma _0^{}h_E^d\big ((\xi _{v_h}^p(x_i)-\xi _{w_h}^p(x_j))\partial _{\varvec{t}}w_h, \partial _{\varvec{t}} z_h\big )_E. \end{aligned}$$

We now remark that for two numbers $a,b\in [0,1]$ we have

$$\begin{aligned} |a^p-b^p| = |a-b|\sum _{l=0}^{p-1}a^lb^{p-1-l}\le p\,|a-b|, \end{aligned}$$

and the term in $E_1$ is bounded using Lemma 1. In fact, from the shape regularity of the mesh sequence there exists $C>0$, independent of *h*, such that for all $E,F\in \mathscr {E}_i, h_F\le C h_E$. Moreover, the number of edges in $\mathscr {E}_i$ is uniformly bounded, independently of *h*. Then, using Cauchy–Schwarz’s inequality and a local trace inequality we arrive at

2.12 $$\begin{aligned}&\sum _{E\in E_1}\gamma _0^{}h_E^d\big ((\xi _{v_h}^p(x_i)-\xi _{w_h}^p(x_i))\partial _{\varvec{t}}w_h, \partial _{\varvec{t}} z_h\big )_E \nonumber \\&\quad \le p\sum _{E\in E_1}\gamma _0^{}h_E^d\big (|\xi _{v_h}(x_i)-\xi _{w_h}(x_i)|\partial _{\varvec{t}}w_h, \partial _{\varvec{t}} z_h\big )_E \nonumber \\&\quad \le p\sum _{E\in E_1}\gamma _0^{}h_E^d\left( 4\frac{\sum _{F\in \mathscr {E}_i}h_F|\partial _{\varvec{t}}(v_h-w_h)|_{F}|}{\sum _{F\in \mathscr {E}_i} h_F(|\partial _{\varvec{t}} v_h|_F|+|\partial _{\varvec{t}} w_h|_F|)}|\partial _{\varvec{t}}w_h|,|\partial _{\varvec{t}}z_h|\right) _E \nonumber \\&\quad \le 4p\,\gamma _0^{}\sum _{E\in E_1}h_E^d\left( \sum _{F\in \mathscr {E}_i}\big |\partial _{\varvec{t}}(v_h-w_h)|_F^{}\big |,|\partial _{\varvec{t}}z_h|\right) _E \nonumber \\&\quad \le C\gamma _0^{}h\,|v_h-w_h|_{1,\Omega }|z_h|_{1,\Omega }. \end{aligned}$$

The sum over $E_2$ is bounded next. First, using (2.12) we get

$$\begin{aligned}&\sum _{E\in E_2} \gamma _0^{}h_E^d\big ((\xi _{v_h}^p(x_i)-\xi _{w_h}^p(x_j))\partial _{\varvec{t}}w_h, \partial _{\varvec{t}} z_h\big )_E \\&\quad = \sum _{E\in E_2} \gamma _0^{}h_E^d\big ((\xi _{v_h}^p(x_i)-\xi _{w_h}^p(x_i))\partial _{\varvec{t}}w_h, \partial _{\varvec{t}} z_h\big )_E \\&\quad \quad + \sum _{E\in E_2} \gamma _0^{}h_E^d\big ((\xi _{w_h}^p(x_i)-\xi _{w_h}^p(x_j))\partial _{\varvec{t}}w_h, \partial _{\varvec{t}} z_h\big )_E\\&\quad \le C\gamma _0^{}h\,|v_h-w_h|_{1,\Omega }|z_h|_{1,\Omega }+\sum _{E\in E_2} \gamma _0^{}h_E^d \big ((\xi _{w_h}^p(x_i)-\xi _{w_h}^p(x_j))\partial _{\varvec{t}}w_h, \partial _{\varvec{t}} z_h\big )_E. \end{aligned}$$

In an analogous way we obtain

$$\begin{aligned}&\sum _{E\in E_2} \gamma _0^{}h_E^d\big ((\xi _{v_h}^p(x_i)-\xi _{w_h}^p(x_j))\partial _{\varvec{t}}w_h, \partial _{\varvec{t}} z_h\big )_E \le C\gamma _0^{}\,h\,|v_h-w_h|_{1,\Omega }|z_h|_{1,\Omega }\\ {}&\quad +\sum _{E\in E_2} \gamma _0^{}h_E^d\big ((\xi _{v_h}^p(x_i)-\xi _{v_h}^p(x_j))\partial _{\varvec{t}}w_h, \partial _{\varvec{t}} z_h\big )_E. \end{aligned}$$

Hence

2.13 $$\begin{aligned}&\sum _{E\in E_2} \gamma _0^{}h_E^d((\xi _{v_h}^p(x_i)-\xi _{w_h}^p(x_j))\partial _{\varvec{t}}w_h, \partial _{\varvec{t}} z_h)_E\nonumber \\&\quad \le C\gamma _0^{}h\,|v_h-w_h|_{1,\Omega }|z_h|_{1,\Omega } \nonumber \\&\quad \quad +\sum _{E\in E_2}\gamma _0^{}h_E^d\min \{ (\xi _{v_h}^p(x_i)-\xi _{v_h}^p(x_j))(\partial _{\varvec{t}}w_h, \partial _{\varvec{t}} z_h)_E,\nonumber \\&\qquad (\xi _{w_h}^p(x_i)-\xi _{w_h}^p(x_j))(\partial _{\varvec{t}}w_h, \partial _{\varvec{t}} z_h)_E\} \nonumber \\&\quad \le C\gamma _0^{}h\,|v_h-w_h|_{1,\Omega }|z_h|_{1,\Omega }, \end{aligned}$$

since the last term in the middle inequality is always non-positive, since by construction, for $E \in E_2$, $\xi _{v_h}^p(x_i) - \xi _{v_h}^p(x_j)\ge 0$ and $\xi _{w_h}^p(x_i) - \xi _{w_h}^p(x_j)\le 0$. The result then follows collecting (2.10)–(2.13). $\square$

#### *Remark 1*

It is worth remarking that a modification of the method can be introduced in such a way that the method becomes linearity preserving on general meshes. This modification is based on the introduction of appropriate weights in the definition of $\xi _{w_h^{}}$. More precisely, instead of its original definition (2.4), we can introduce the following modified one: for $w_h^{}\in \mathcal {V}_h^{}$ and any internal node $x_i^{}$

$$\begin{aligned} \xi _{w_h^{}}(x_i^{}):= \left\{ \begin{array}{ll} \frac{\left| \sum \nolimits _{j\in S_{i}} \beta _{ij}(w_h^{}(x_j^{})-w_h^{}(x_i^{}))\right| }{\sum \nolimits _{j\in S_{i}}\beta _{ij}|w_h^{}(x_i^{})- w_h^{}(x_j^{})|} &{}\quad \text {if}~ \sum \nolimits _{j\in S_{i}}\beta _{ij}|w_h^{}(x_j^{})-w_h^{}(x_i^{})|\ne 0,\\ 0 &{} \quad \text {otherwise}. \end{array} \right. \end{aligned}$$

The coefficients $\beta _{ij}^{}$ are designed in such a way that they satisfy the linearirty preservation property. Denoting $\varvec{\tau }_{ij}^{} = x_j-x_i$, this condition reads

$$\begin{aligned} \forall v\in \mathbb {P}_1(\Omega _i)\;\; \sum _{j\in S_i}\beta _{ij}^{}\big (v(x_j)-v(x_i)\big ) = \sum _{j\in S_i}\beta _{ij}^{}\nabla v\cdot \varvec{\tau }_{ij}^{} = \nabla v\cdot \left( \sum _{j\in S_i}\beta _{ij}^{}\varvec{\tau }_{ij}^{} \right) = 0, \end{aligned}$$

which is equivalent to imposing

2.14 $$\begin{aligned} \sum _{j\in S_i}\beta _{ij}^{}\varvec{\tau }_{ij}^{} = \mathbf {0}. \end{aligned}$$

The Eq. (2.14) is a first restriction that the coefficients have to satisfy. A further restriction on $\beta _{ij}^{}$ is their strict positivity. Then, we impose

2.15 $$\begin{aligned} \beta _{ij}^{}\ge C_0>0, \end{aligned}$$

where the value of $C_0$ is of no great importance. Finally, in case the mesh is symetric with respect to its interior nodes, then $\beta _{ij}^{}=1$ for all *i*, *j* should be an acceptable (and preferred) solution. Then, we find $\beta _{ij}^{}$ as the solution of the following problem: for all internal node $x_i^{}$, find

2.16 $$\begin{aligned} \big (\beta _{ij}^{}\big )_{j\in S_i^{}}=\text {argmin}\left\{ \sum _{j\in S_{i^{}}}|\delta _{ij}^{}-1|^2:\{\delta _{ij}^{}\} \;\text {satisfies the restrictions}\;(2.14), (2.15) \right\} . \end{aligned}$$

The same results that are presented for the original definition of $\xi$ in (2.4) can be obtained for the present modification. For simplicity of the presentation, and also to avoid the computational complexity of solving the constrained optimisation problem (2.16), we have preferred to use in the rest of the paper the original definition (2.4).

### Solvability of the discrete problem

This section is devoted to analyse the existence of solutions for (2.2). It is interesting to remark that, thanks to the Lipschitz continuity of $d_h(\cdot ;\cdot ,\cdot )$, the solution can be proved to be unique in the diffusion-dominated regime.

#### **Lemma 3**

Let $T_h:\mathcal {V}_h^0 \rightarrow [\mathcal {V}_h^0]'$ be the operator defined by

2.17 $$\begin{aligned}{}[T_h z_h, v_h] = \,a_h(z_h + u_{bh} ; v_h) - (f,v_h)_\Omega , \quad z_h, v_h \in \mathcal {V}_h^0, \end{aligned}$$

where $[\cdot ,\cdot ]$ denotes the duality pairing between $\mathcal {V}_h^0$ and its dual. Then,

2.18 $$\begin{aligned}{}[T_h z_h, z_h] \ge c_1 |z_h|_{1,\Omega }^2 - c_2 (\Vert u_{bh}\Vert _{1,{\Omega }}^2 + \Vert f\Vert _{0,{\Omega }}^2), \end{aligned}$$

where $c_1, c_2$ are positive constants independent of $z_h, f$, and *g*.

#### *Proof*

For this proof only, we will consider constants $C>0$ that may depend on the physical coefficients. From the definition of *a* it follows that

2.19 $$\begin{aligned} a(z_h,z_h)=\,\varepsilon \,|z_h|^2_{1,\Omega }+(\sigma z_h,z_h)\ge \varepsilon \,|z_h|^2_{1,\Omega }. \end{aligned}$$

Moreover, the definition of $d_h(\cdot ;\cdot ,\cdot )$ and the fact that $0\le \alpha _E^{}(z_h + u_{bh})$ give

2.20 $$\begin{aligned} d_h(z_h + u_{bh} ; z_h, z_h) = \sum _{E\in \mathscr {E}_h}\,\gamma _0^{} \, h_E^d\, \alpha _E^{}(z_h + u_{bh}) \Vert \partial _{\varvec{t}} z_h \Vert _{0,E}^2 \ge 0. \end{aligned}$$

Then, the definition of the operator $T_h$ gives

2.21 $$\begin{aligned}{}[T_h z_h, z_h] \ge \varepsilon |z_h|_{1,\Omega }^2 + a(u_{bh}, z_h) + d_h(z_h + u_{bh} ; u_{bh}, z_h) - (f,z_h)_\Omega . \end{aligned}$$

Next, the Cauchy–Schwarz and Poincaré inequalities lead to the following bound

2.22 $$\begin{aligned} a(u_{bh}, z_h)|&= \left| \varepsilon (\nabla u_{bh}, \nabla z_h)_\Omega + (\varvec{b}\cdot \nabla u_{bh}, z_h)_\Omega +(\sigma u_{bh},z_h)^{}\right| \nonumber \\&\le \varepsilon \, |u_{bh}|_{1,{\Omega }} |z_h|_{1,{\Omega }} + \Vert \varvec{b}\Vert _{\infty , \Omega } \Vert u_{bh}\Vert _{1,\Omega } \Vert z_h\Vert _{0,\Omega } +C\sigma \,\Vert u_{bh}\Vert _{0,\Omega }^{}\Vert z_h\Vert _{0,\Omega }^{} \nonumber \\&\le C \Vert u_{bh}\Vert _{1,{\Omega }} | z_h|_{1,\Omega }. \end{aligned}$$

In addition, using the shape regularity of the mesh sequence, $\alpha _E^{}(\cdot )\le 1$, and the local trace inequality, we arrive at

2.23 $$\begin{aligned} |d_h(z_h + u_{bh} ; u_ {bh}, z_h) |&= \sum _{E\in \mathscr {E}_h}\, \gamma _0^{}\, h_E^d \, \alpha _E^{}(z_h + u_{bh}) (\partial _{\varvec{t}} u_{bh} , \partial _{\varvec{t}} z_h)_E \nonumber \\&\le \sum _{E\in \mathscr {E}_h}\, \gamma _0^{}\, h_E^d\, \Vert \partial _{\varvec{t}} u_{bh}\Vert _{0,E} \Vert \partial _{\varvec{t}} z_h \Vert _{0,E} \nonumber \\&\le C \, h \,|u_{bh}|_{1,\Omega } | z_h|_{1,\Omega } . \end{aligned}$$

We can thus conclude that

$$\begin{aligned}{}[T_h z_h, z_h] \ge \varepsilon |z_h|_{1,\Omega }^2 - C\,\Vert u_{bh}\Vert _{1,\Omega } | z_h|_{1,\Omega } - \Vert f\Vert _{0,{\Omega }} \Vert z_h\Vert _{0,\Omega }. \end{aligned}$$

The claimed result arises by applying the Poincaré and Young inequalities to the last relation. $\square$

The solvability of the nonlinear problem (2.2) appears as a consequence of the above result and Brower’s fixed point theorem.

#### **Theorem 1**

The discrete problem (2.2) has at least one solution. Moreover, if $C_\mathrm{lip}\gamma _0^{}\,h < \varepsilon$, where $C_\mathrm{lip}$ is the constant from Lemma 2, then the solution is unique.

#### *Proof*

First, since the bilinear form $a(\cdot ,\cdot )$ is continuous, and $d_h(\cdot ;\cdot ,\cdot )$ is Lipschitz continuous, then the operator $T_h$ is Lipschitz continuous. Next, in view of (2.18), for any $z_h\in \mathcal {V}_h^0$ such that

$$\begin{aligned} |z_h|^2_{1,\Omega } = 2\frac{c_2(\Vert u_{bh}\Vert _{1,\Omega }^2+\Vert f\Vert _{0,\Omega }^2)}{c_1}, \end{aligned}$$

Lemma 3 gives

2.24 $$\begin{aligned}{}[T_h z_h, z_h] = c_2(\Vert u_{bh}\Vert _{0,\Omega }^2 + \Vert f\Vert _{0,\Omega }^2) >0. \end{aligned}$$

Then, using a consequence of Brower’s fixed point Theorem (see [11, Corollary 1.1, Ch. IV]), there exists $\tilde{v}_h\in \mathcal {V}_h^0$ such that $T_h(\tilde{v}_h)=0$. Hence, $u_h:=\tilde{v}_h+u_{bh}$ solves (2.2).

In order to prove uniqueness, let $u_h^1, u_h^2$ be two solutions of (2.2). Then, using (2.2) for both solutions, denoting $\tilde{e}^{}_h:=u_h^1-u_h^2$, and using the Lipschitz continuity of $d_h(\cdot ;\cdot ,\cdot )$, we obtain

2.25 $$\begin{aligned} \varepsilon \,|\tilde{e}^{}_h|^2_{1,\Omega } \le a(\tilde{e}^{}_h,\tilde{e}^{}_h) = -d_h(u_h^1;u_h^1,\tilde{e}^{}_h)+d_h(u_h^2;u_h^2,\tilde{e}^{}_h) \le C_\mathrm{lip}\gamma _0^{}h\,|\tilde{e}^{}_h|^2_{1,\Omega }. \end{aligned}$$

This leads to

2.26 $$\begin{aligned} (\varepsilon -C_\mathrm{lip}\gamma _0^{}h)\,|\tilde{e}^{}_h|^2_{1,\Omega } \le 0, \end{aligned}$$

which, using that $\tilde{e}^{}_h\in H^1_0(\Omega )$, finishes the proof. $\square$

### The discrete maximum principle

This section is devoted to prove that method (2.2) preserves positivity. For this, we will impose the following geometric hypothesis on the mesh. This hypothesis can be tracked back to [22], and in two space dimensions it reduces to impose that the mesh is Delaunay.

#### **Assumption 1**

(*Hypothesis of Xu and Zikatanov, cf.* [22]) For every internal edge $E\in \mathscr {E}_h$ with end points $x_i$ and $x_j$ the following inequality holds

2.27 $$\begin{aligned} \frac{1}{d(d-1)}\sum _{K\in \omega _E}|\omega _{ij}^K|\cot (\theta _{ij}^K)\ge 0, \end{aligned}$$

where $\theta _{ij}^K$ is the angle between the two facets in *K* opposite to $x_i$ and $x_j$ (denoted by $F_{i,K}$ and $F_{j,K}$, respectively), and $\omega ^K_{ij}$ is the $(d-2)$-dimensional simplex $F_{i,K} \cap F_{j,K}$ opposite to the edge *E*.

We now introduce the discrete analogue of the maximum principle. This definition is related to the one from [7], and it leads to results which are, essentially, identical to those from that reference.

#### **Definition 2**

(*DMP*) The semilinear form $a_h(\cdot ;\cdot )$ is said to satisfy the *strong DMP property* if the following holds: for all $u_h\in \mathcal {V}_h$ and for all interior vertices $x_i$, if $u_h$ is locally minimal (resp. maximal) on the vertex $x_i$ over the macro-element $\Omega _i$, then there exist negative quantites $( c_E)_{E\in \mathscr {E}_i}$ such that

2.28 $$\begin{aligned} a_h(u_h;\psi _i)\le \sum _{E\in \mathscr {E}_i} c_E\big |\partial _{\varvec{t}}u_h|_{E}\big |, \end{aligned}$$

[resp. $a_h(u_h;\psi _i)\ge -\sum _{E\in \mathscr {E}_i} c_E\big |\partial _{\varvec{t}}u_h|_{E}\big |$]. Furthermore, we will say that the semilinear form satisfies the weak DMP property, related to local minima, if (2.28) holds only under the additional assumption that the local minimum above is supposed to be negative.

A direct consequence of this definition is the following result analoguous to that of [7, Proposition 2.5]. We reproduce the proof here for the reader’s convenience.

#### **Lemma 4**

Assume that the semilinear form $a_h(\cdot ;\cdot )$ satisfies the DMP property. Assume that $u_h\in \mathcal {V}_h$ solves (2.2) and that $f\ge 0$. Then $u_h$ reaches its minimum on the boundary $\partial \Omega$ and for the weak DMP-property, if $g\ge 0$, then $u_h \ge 0$ in $\Omega$.

#### *Proof*

Assume that the DMP is satisfied and $u_h$ reaches its minimum in an interior vertex $x_i$. Since $a_h(\cdot ;\cdot )$ satisfies (2.28), $u_h$ is constant over $\Omega _i$, implying that the minimum is taken in all vertices $x_j \in \Omega _i$. Repeating the argument we eventually deduce that the minimum is reached on the boundary. $\square$

The following result states the DMP for (2.2).

#### **Theorem 2**

Let us suppose that the mesh $\mathscr {T}_h$ satisfies Assumption 1, and that the parameter $\gamma _0^{}$ is large enough. Then, the semilinear form $a_h(\cdot ;\cdot )$ satisfies the weak DMP property for $\sigma >0$ and the strong DMP-property for $\sigma =0$.

#### *Proof*

Let us suppose that $u_h$ has a negative local minimum at an interior node $x_i$. Then, $\alpha _E^{}(u_h)=1$ for all $E\in \mathscr {E}_i$, which gives

2.29 $$\begin{aligned} a_h(u_h;\psi _i)= & {} (\sigma u_h,\psi _i)_\Omega +\varepsilon (\nabla u_h,\nabla \psi _i)_\Omega + (\varvec{b}\cdot \nabla u_h,\psi _i)_\Omega \nonumber \\&+\sum _{E\in \mathscr {E}_i}\gamma _0^{}h_E^d(\partial _{\varvec{t}}u_h,\partial _{\varvec{t}} \psi _i)_E. \end{aligned}$$

We will analyse the expression above term-by-term. First, if $u_h\le 0$ in the support of $\psi _i$, then $(\sigma u_h,\psi _i)_\Omega \le 0$. Let us suppose now that $u_h$ changes sign in the support of $\psi _i$, and let $K\in \Omega _i$ be an element in which $u_h$ changes sign. Let $x_k$ be a node in *K* such that $u_h(x_k)\ge 0$, and let $E_{ik}^{}$ be the edge connecting these two nodes. Then, using the Cauchy–Schwarz inequality, a Poincaré inequality in *K*, and the shape regularity of the mesh sequence, we arrive at

$$\begin{aligned} (\sigma u_h,\psi _i)_K&\le \sigma \,\Vert u_h^{}\Vert ^{}_{0,K}\Vert \psi _i^{}\Vert ^{}_{0,K}\\&\le C\sigma \,h_K^{\frac{d}{2}}\Vert u_h^{}\Vert ^{}_{0,K}\\&\le C\sigma \,h_K^{d}\,h_{E_{ik}}^{}\big |\partial _{\varvec{t}}u_h|_{E_{ik}}\big |. \end{aligned}$$

Then, adding up over all $K\in \Omega _i$ and using the shape regularity of the mesh sequence we obtain

2.30 $$\begin{aligned} (\sigma u_h,\psi _i)_\Omega \le C_0 \sigma \sum _{E \in \mathcal {E}_i} h^{d+1}_E |\partial _\mathbf{t} u_h|_E|. \end{aligned}$$

Also, as in [7] (see also [21]), Assumption 1 on the mesh leads to

2.31 $$\begin{aligned} \varepsilon (\nabla u_h,\nabla \psi _i)_\Omega \le 0. \end{aligned}$$

Moreover $\sum _{j=1}^N\psi _j=1$ gives $\sum _{j\in S_i} (\varvec{b}\cdot \nabla \psi _j,\psi _i)_\Omega =0$, and then

2.32 $$\begin{aligned} (\varvec{b}\cdot \nabla u_h, \psi _i)_\Omega&= \sum _{j\in S_i}(\varvec{b}\cdot \nabla \psi _j, \psi _i)_\Omega u_h(x_j) +(\varvec{b}\cdot \nabla \psi _i, \psi _i)_\Omega u_h(x_i) \nonumber \\&= \sum _{j\in S_i}(\varvec{b}\cdot \nabla \psi _j, \psi _i)_\Omega \big ( u_h(x_j)-u_h(x_i)\big ) \nonumber \\&= \sum _{E\in \mathscr {E}_i}(\varvec{b}\cdot \nabla \psi _j, \psi _i)_\Omega h_E \big |\partial _{\varvec{t}} u_h|_E\big |, \end{aligned}$$

which, using the shape regularity of the mesh sequence gives

2.33 $$\begin{aligned} (\varvec{b}\cdot \nabla u_h, \psi _i)_\Omega \le \sum _{E\in \mathscr {E}_i} C_1\Vert \varvec{b}\Vert _{\infty ,E}h_E^d|\partial _{\varvec{t}} u_h|_E|. \end{aligned}$$

Finally, since $u_h(x_i)$ is a local minimum, then in every $E\in \mathscr {E}_i$, $\partial _{\varvec{t}} u_h$ and $\partial _{\varvec{t}} \psi _i$ have different signs (independently of the orientation of the tangential vector in *E*), which gives

2.34 $$\begin{aligned} \sum _{E\in \mathscr {E}_i}\gamma _0^{}h_E^d(\partial _{\varvec{t}}u_h,\partial _{\varvec{t}} \psi _i)_E = -\sum _{E\in \mathscr {E}_i}\gamma _0^{}h_E^d\big |\partial _{\varvec{t}} u_h|_E\big |. \end{aligned}$$

Hence, gathering all the above computations, we arrive at

2.35 $$\begin{aligned} a_h(u_h;\psi _i)\le -\sum _{E\in \mathscr {E}_i}(\gamma _0^{}- C_0 \sigma h_E- C_1\Vert \varvec{b}\Vert _{\infty ,E})h_E^d\big |\partial _{\varvec{t}} u_h|_E\big |, \end{aligned}$$

and the result follows assuming that $\gamma _0^{}> C_0 \sigma h_E + C_1\Vert \varvec{b}\Vert _{\infty ,E}$. Finally, we notice that if $\sigma =0$ then the sign of the strict minimum is irrelevant, which proves the strong DMP property. $\square$

#### *Remark 2*

It is interesting to remark that the hypothesis on the meshes of the triangulation can be avoided if the problem is supposed to be strongly convection-dominated. In fact, following analogous steps to those used to prove (2.32) we can arrive at

2.36 $$\begin{aligned} \varepsilon (\nabla u_h,\nabla \psi _i)_\Omega = \varepsilon \sum _{E\in \mathscr {E}_i}(\nabla \psi _j,\nabla \psi _i)_\Omega h_E|\partial _{\varvec{t}}u_h| \le \sum _{E\in \mathscr {E}_i}{C_2}\varepsilon h_E^{d-1} |\partial _{\varvec{t}}u_h|. \end{aligned}$$

Replacing this into the steps leading to (2.35) gives

2.37 $$\begin{aligned} a_h(u_h;\psi _i)\le -\sum _{E\in \mathscr {E}_i}(\gamma _0^{}-C_0 \sigma h_E - C_1\Vert \varvec{b}\Vert _{\infty ,E} - C_2\varepsilon h_E^{-1})h_E^d|\partial _{\varvec{t}} u_h|, \end{aligned}$$

and the proof follows by assuming that $\gamma _0^{}> C_0 \sigma h_E +C_1\Vert \varvec{b}\Vert _{\infty ,E} + C_2\varepsilon h_E^{-1}$.

The last result is only interesting if $\varepsilon h_E^{-1}$ stays bounded, which means this is applicable only in the case the problem is highly convection-dominated. In this sense, the method proposed in this work can be applied to scalar conservation laws, regardless of the geometrical impositions on the mesh. Similar results have been obtained recently in [12, 13].

## Convergence

The error will be analysed using the following norm:

3.1 $$\begin{aligned} \Vert v_h\Vert _h^2:= \sigma \Vert v_h\Vert ^2_{0,\Omega }+\varepsilon \,|v_h|^2_{1,\Omega }+d_h(u_h;v_h,v_h). \end{aligned}$$

This norm is not only mesh-dependent, but also depends on the discrete solution. The inclusion of the last term in it is made mostly for convenience, but the fact that it controls the usual $H^1(\Omega )$-norm (weighted by physical coefficients) guarantees that the convergence of the method is valid with respect to the standard norm as well. As usual, the error $e:=u-u_h$ is split as follows

3.2 $$\begin{aligned} e = u- u_h = (u- i_h^{}u) + (i_h^{} u - u_h):= \rho _h + e_h, \end{aligned}$$

where $i_h^{}:C^0(\overline{\Omega })\cap H^1_0(\Omega )\rightarrow \mathcal {V}^0_h$ stands for the Clément interpolation operator. Using standard interpolation estimates (see [8]), the fact that $\alpha _E^{}(\cdot )\le 1$, and the shape regularity of the mesh sequence, the following bound for $\rho _h$ follows:

3.3 $$\begin{aligned} \Vert \rho _h\Vert _h^{} \le C( \varepsilon ^{\frac{1}{2}} + \sigma ^{\frac{1}{2}} h + \gamma _0^{}h^{\frac{1}{2}} )\,h\, \Vert u\Vert _{2,\Omega }. \end{aligned}$$

The next result states a bound for $e_h$.

### **Lemma 5**

Let us suppose $u\in H^2(\Omega )\cap H^1_0(\Omega )$. Then, there exists $C>0$, independent of *h* and $\varepsilon$, such that

3.4 $$\begin{aligned} \Vert e_h\Vert _h^{} \le C\,\big (\varepsilon +\sigma ^{-1}\{\Vert \varvec{b}\Vert ^2_{\infty ,\Omega }+\sigma ^2\}\big )^{\frac{1}{2}}h\Vert u\Vert _{2,\Omega }+Ch^{\frac{1}{2}}\,\Vert u\Vert _{1,\Omega }. \end{aligned}$$

### *Proof*

First, from the definition of *a* and $d_h$ we get

3.5 $$\begin{aligned} \Vert e_h\Vert ^2_h =&\, a(e_h,e_h)+d_h(u_h;e_h,e_h) \nonumber \\ =&\,a(i_h^{}u,e_h)-\{ a(u_h,e_h)+d_h(u_h;u_h,e_h)\}+d_h(u_h;i_h^{}u,e_h) \nonumber \\ =&\,-a(\rho _h,e_h)+d_h(u_h;i_h^{}u,e_h). \end{aligned}$$

Next, the continuity of *a* gives

3.6 $$\begin{aligned} a(\rho _h,e_h)&\le (\sigma \Vert \rho _h\Vert ^2_{0,\Omega }+[\varepsilon +\sigma ^{-1}\Vert \varvec{b}\Vert _{\infty ,\Omega }^2] \,|\rho _h|^2_{1,\Omega })^{\frac{1}{2}} \Vert e_h\Vert _h\, \nonumber \\&\le C( \varepsilon ^{\frac{1}{2}} + \sigma ^{-1/2} \Vert \varvec{b}\Vert _{\infty ,\Omega }+\sigma ^{\frac{1}{2}} h )\,h\, \Vert u\Vert _{2,\Omega }\Vert e_h\Vert _h. \end{aligned}$$

Moreover, since $d_h(u_h;\cdot ,\cdot )$ is a symmetric positive semi-definite bilinear form it satisfies Cauchy–Schwarz’s inequality, which gives

3.7 $$\begin{aligned} d_h(u_h;i_h^{}u,e_h)\le d_h(u_h;i_h^{}u,i_h^{}u)^{\frac{1}{2}}d_h(u_h;e_h,e_h)^{\frac{1}{2}}\le d_h(u_h;i_h^{}u,i_h^{}u)^{\frac{1}{2}} \Vert e_h\Vert _h. \end{aligned}$$

Then, inserting (3.6) and (3.7) into (3.5), and using Young’s inequality, we arrive at

3.8 $$\begin{aligned} \Vert e_h\Vert ^2_h \le C( \varepsilon ^{\frac{1}{2}} + \sigma ^{-1/2} \Vert \varvec{b}\Vert _{\infty ,\Omega }+\sigma ^{\frac{1}{2}} h )^2\,h^2\, \Vert u\Vert ^2_{2,\Omega } +C\,d_h(u_h;i_h^{}u,i_h^{}u). \end{aligned}$$

It only remains to bound the consistency error $d_h(u_h;i_h^{}u,i_h^{}u)$ in (3.8). The definition of $d_h(\cdot ;\cdot ,\cdot )$, $\alpha _E^{}(u_h)\le 1$, a local trace inequality, the shape regularity of the mesh sequence, and the $H^1(\Omega )$-stability of $i_h^{}$, give

3.9 $$\begin{aligned} d_h(u_h;i_h^{}u,i_h^{}u)= & {} \sum _{E\in \mathscr {E}_h}\gamma _0^{}h_E^d\alpha _E^{}(u_h)\Vert \partial _{\varvec{t}}i_h^{}u\Vert _{0,E}^2\nonumber \\\le & {} \gamma _0^{}h\sum _{E\in \mathscr {E}_h}h_E^{d-1}\,\Vert \partial _{\varvec{t}}i_h^{}u\Vert ^2_{0,E}\le Ch\,\Vert u\Vert ^2_{1,\Omega }. \end{aligned}$$

Then, the result arises inserting (3.9) into (3.8). $\square$

Collecting (3.3) and Lemma 5 we then obtain the following error estimate for (2.2).

### **Theorem 3**

Let us suppose $u\in H^2(\Omega )\cap H^1_0(\Omega )$. Then, there exists $C>0$, independent of *h* and $\varepsilon$, such that

3.10 $$\begin{aligned} \Vert e\Vert _h^{} \le C\,\big (\varepsilon +\sigma ^{-1}\{\Vert \varvec{b}\Vert ^2_{\infty ,\Omega }+\sigma ^2\}\big )^{\frac{1}{2}}h\Vert u\Vert _{2,\Omega }+Ch^{\frac{1}{2}}\,\Vert u\Vert _{1,\Omega }. \end{aligned}$$

The following result states that for meshes which are symmetric with respect to their interior nodes, the method converges with a higher order. This result’s main interest lies in the diffusion dominated regime, due to the factor $\varepsilon ^{-\frac{1}{2}}$ present in the estimate. The combination of Lipschitz continuity and linearity preservation seems to be novel, and that is why we do detail it now.

### **Theorem 4**

Let us suppose $u\in H^2(\Omega )\cap H^1_0(\Omega )$ and that the mesh is symmetric with respect to its internal nodes. Then, there exists $C>0$, independent of *h* and $\varepsilon$, such that

3.11 $$\begin{aligned} \Vert e\Vert _h^{} \le C\,\big (\varepsilon +\sigma ^{-1}\{\Vert \varvec{b}\Vert ^2_{\infty ,\Omega }+\sigma ^2\}\big )^{\frac{1}{2}}h\Vert u\Vert _{2,\Omega }+C\frac{h}{\sqrt{\varepsilon }}\, \Vert u\Vert _{1,\Omega }. \end{aligned}$$

### *Proof*

It is enough to bound the consistency error $d(u_h;i_h^{}u,i_h^{}u)$. We have

3.12 $$\begin{aligned} d_h(u_h;i_h^{}u,i_h^{}u)&=\{ d_h(u_h;i_h^{}u,i_h^{}u)-d_h(i_h^{}u;i_h^{}u,i_h^{}u)\}+d_h(i_h^{}u;i_h^{}u,i_h^{}u)\nonumber \\&=:\mathrm {I}+\mathrm {II}. \end{aligned}$$

The first term is bounded as in the proof of Lemma 2. In fact, in that proof, the bound for the second term in (2.10) leads to the following

3.13 $$\begin{aligned} \mathrm {I}&=\sum _{E\in \mathscr {E}_h}(\alpha _E^{}(u_h)-\alpha _E(i_h^{}u))\gamma _0^{}h_E^d(\partial _{\varvec{t}} i_h^{}u,\partial _{\varvec{t}} i_h^{}u)_E\nonumber \\&\le Ch|u_h-i_h^{}u|_{1,\Omega }^{}|i_h^{}u|^{}_{1,\Omega }\nonumber \\&\le \frac{\varepsilon }{2}\,|u_h-i_h^{}u|_{1,\Omega }^2+C\frac{h^2}{\varepsilon }\,\Vert u\Vert ^2_{1,\Omega }, \end{aligned}$$

where we have also used the $H^1(\Omega )$-stability of $i_h^{}$. To bound $\mathrm {II}$ we use the linearity preservation and the Lipschitz continuity of $d_h(\cdot ;\cdot ,\cdot )$. More precisely, for a given $E\in \mathscr {E}_h$ we introduce the function $i_E^{}u\in \mathbb {P}_1(\omega _E)$ as the unique solution of the problem

3.14 $$\begin{aligned} (\nabla i_E^{}u,\nabla \psi )_{\omega _E^{}}&=(\nabla u,\nabla \psi )_{\omega _E^{}}\quad \forall \psi \in \mathbb {P}_1(\omega _E^{}),\\ (i_E^{}u,1)_{\omega _E^{}}&=(u,1)_{\omega _E^{}}.\nonumber \end{aligned}$$

Using standard finite element approximation results (see [8]), $i_E^{}u$ satisfies

3.15 $$\begin{aligned} |u-i_E^{}u|^{}_{1,\omega _E^{}}\le Ch_E^{}|u|^{}_{2,\omega _E^{}}. \end{aligned}$$

Since the mesh is symmetric with respect to its internal nodes, $\alpha _E^{}(i_E^{}u)=0$. Then, proceeding as in the bound for $\mathrm {I}$ we obtain

3.16 $$\begin{aligned} \mathrm {II}&= \sum _{E\in \mathscr {E}_h} (\alpha _E^{}(i_h^{}u)-\alpha _E^{}(i_E^{}u))\gamma _0^{}h_E^d\, (\partial _{\varvec{t}} i_h^{}u,\partial _{\varvec{t}} i_h^{}u)_E\nonumber \\&\le Ch\,\left\{ \sum _{E\in \mathscr {E}_h}|i_h^{}u-i_E^{}u|^2_{1,\omega _E^{}}\right\} ^{\frac{1}{2}}|i_h^{}u|_{1,\Omega }^{}\nonumber \\&\le Ch^2|u|_{2,\Omega }^{}\Vert u\Vert _{1,\Omega }^{}. \end{aligned}$$

Then, inserting (3.13) and (3.16) into (3.12) we obtain

3.17 $$\begin{aligned} d_h(u_h;i_h^{}u,i_h^{}u)\le \frac{\varepsilon }{2}\,|u_h-i_h^{}u|_{1,\Omega }^2+C\frac{h^2}{\varepsilon }\,\Vert u\Vert ^2_{1,\Omega }+Ch^2|u|_{2,\Omega }^{}\Vert u\Vert _{1,\Omega }^{}, \end{aligned}$$

and the result follows by rearranging terms. $\square$

## A link to algebraic flux correction schemes

Method (2.2) has been presented having as motivation the study of the effect of adding edge-based diffusion into the equations to impose the discrete maximum principle. Another family of methods that are built with the same purpose is the AFC schemes. This section is devoted to study the relationship between the two approaches, and that is why we now summarise the main building principles of AFC schemes.

The starting point of an algebraic flux-correction scheme is a discretisation of the convection–diffusion–reaction equation which leads to the linear system

4.1 $$\begin{aligned} \mathbb {A}\mathrm {U}=\mathbb {G}, \end{aligned}$$

where $\mathbb {A}=(a_{ij})_{i,j=1}^N$, $\mathrm {U}=\{u_h(x_i)\}_{i=1}^N$ and $\mathbb {G}=\{g_i\}_{i=1}^N$. The first step of these schemes is to identify which parts of the system matrix $\mathbb {A}$ are responsible for the violation of the discrete maximum principle. To achieve this, the diffusion matrix $\mathbb {D}=(d_{ij})_{i,j=1}^N$ is built, where

$$\begin{aligned} d_{ij}=d_{ji}=-\max \{ a_{ij},0,a_{ji}\}\quad \forall i\not = j\,\quad d_{ii}=-\sum _{j\not = i} d_{ij}. \end{aligned}$$

Adding $\mathbb {D}\mathrm{U}$ both sides of (4.1) we obtain

4.2 $$\begin{aligned} {\tilde{\mathbb {A}}}\mathrm{U}=\mathbb {G}+\mathbb {D}\mathrm{U}, \end{aligned}$$

where $\tilde{\mathbb {A}}:=\mathbb {A}+\mathbb {D}$. Since the matrix $\tilde{\mathbb {A}}$ fullfils the hypothesis to guarantee the discrete maximum principle, then the oscillations that appear in a non-stabilised discretisation (4.1) are due to the right-hand side. This is why the right-hand side is now rewritten. Using that the row-sums of $\mathbb {D}$ are zero, then

$$\begin{aligned} (\mathbb {D}\mathrm {U})_i=\sum _{j\not = i}f_{ij}\quad \text {where}\quad f_{ij}=d_{ij}(u_h(x_j)-u_h(x_i)). \end{aligned}$$

The quantities $f_{ij}$ are called *fluxes*. Then, the AFC schemes are based on introducing limiters $\alpha _{ij}(u_h)$ such that $\alpha _{ij}\in [0,1]$, $\alpha _{ij}=\alpha _{ji}$, and $\alpha _{ij}=1$ if $x_i$ and $x_j$ are both Dirichlet nodes. Then, after introducing these limiters, the method reads as follows:

4.3 $$\begin{aligned} \mathbb {A}\mathrm {U}_i+\sum _{i, j=1}^N(1-\alpha _{ij}(u_h)) d_{ij}\,(u_h(x_j)-u_h(x_i))= g_i. \end{aligned}$$

The most popular limiters in practice are Zalesak’s limiters (see, Refs. [15–17, 23], and the recent review [18] for examples). The analysis of these methods for a class of limiters that includes the Zalesak one has been carried out recently in [2, 3]. In particular, in [2] an $O(h^{\frac{1}{2}})$ convergence rate was proved for the case in which the mesh used satisfies Assumption 1. In the case of meshes that do not satisfy this assumption, then no convergence can be proved, unless some appropriate modifications are done to the algorithm. This result is optimal, as the numerical results in [2] show.

Following [2], Eq. (4.3) can be written as the following weak problem: find $u_h\in \mathcal {V}_h$ such that $u_h-u_{bh}\in \mathcal {V}_h^0$, and

4.4 $$\begin{aligned} a(u_h,v_h)+\tilde{d}_h(u_h;u_h,v_h)=(f,v_h)_\Omega \quad \forall v_h\in \mathcal {V}_h^0, \end{aligned}$$

where the nonlinear form $\tilde{d}_h(\cdot ;\cdot ,\cdot )$ is given by

4.5 $$\begin{aligned} \tilde{d}_h(u_h;u_h,v_h)=\sum _{i, j=1}^N(1-\alpha _{ij}(u_h)) d_{ij}\,(u_h(x_j)-u_h(x_i))v_h(x_i). \end{aligned}$$

Next, to link this to the method analysed in the last sections, we use the symmetry of $\mathbb {D}$, and of the limiters $\alpha _{ij}=\alpha _{ji}$, and a simple calculation gives:

4.6 $$\begin{aligned} \tilde{d}_h(u_h;u_h,v_h)&=\sum _{i>j}(1-\alpha _{ij}(u_h)) d_{ij}\,(u_h(x_j)-u_h(x_i))v_h(x_i) \nonumber \\&\quad + \sum _{i<j}(1-\alpha _{ij}(u_h)) d_{ij}\,(u_h(x_j)-u_h(x_i))v_h(x_i) \nonumber \\&= \sum _{i>j}(1-\alpha _{ij}(u_h)) d_{ij}\,(u_h(x_j)-u_h(x_i))v_h(x_i)\nonumber \\&\quad + \sum _{i>j}(1-\alpha _{ji}(u_h)) d_{ji}\,(u_h(x_i)-u_h(x_j))v_h(x_j) \nonumber \\&= \sum _{i>j}(1-\alpha _{ij}(u_h)) d_{ij}\,(u_h(x_j)-u_h(x_i))(v_h(x_i)-v_h(x_j)). \end{aligned}$$

Then, since $d_{ij}=0$ for $j\not \in S_i$, $\tilde{d}_h(\cdot ;\cdot ,\cdot )$ can be rewritten as

4.7 $$\begin{aligned} \tilde{d}_h(u_h;u_h,v_h) = \sum _{E\in \mathscr {E}_h} (1-\alpha _{ij}(u_h)) |d_{ij}|h_E\,(\partial _{\varvec{t}}u_h,\partial _{\varvec{t}}v_h)_E, \end{aligned}$$

where we have adopted the convention that an edge $E\in \mathscr {E}_h$ has endpoints $x_i$ and $x_j$, and used that $\alpha _{ij}=1$ for edges included in the Dirichlet boundary.

Method (2.2) then appears as an algebraic flux-correction scheme, with a different definition of the limiters. Indeed comparing (2.2) with (4.7) we get the equivalent AFC scheme if we choose $\alpha _{ij}(u_h)$ such that

$$\begin{aligned} (1-\alpha _{ij}(u_h)) |d_{ij}|h_E = \gamma _0^{}\, h_E^d \, \alpha _E^{}(u_h). \end{aligned}$$

The new definition of the limiters made it possible to write some convergence and existence results, also present in [2], in a more precise way, and improve in some of them. In particular, the new limiters make it possible to prove convergence for general meshes, as well as to prove uniqueness of solutions and optimal convergence in the diffusion dominated regime.

## Numerical results

In this section we present three sets of numerical results for bi-dimensional problems. All three cases are set in $\Omega =(0,1)^2$. The nonlinear system (2.2) has been solved using the following fixed-point algorithm with damping: starting with the Galerkin solution $u_h^0$, then compute a sequence $\{ u_h^k\}$ defined by

5.1 $$\begin{aligned} u_h^{k+1}=u_h^k+\omega \,(\tilde{u}_h^{k+1}-u_h^k)\quad k=0,1,2,\ldots , \end{aligned}$$

where $\omega \in (0,1)$ is a damping parameter, and $\tilde{u}_h^{k+1}$ solves: $\tilde{u}_h^{k+1}-u_{bh}\in \mathcal {V}_h^0$, and

5.2 $$\begin{aligned} a(\tilde{u}_h^{k+1},v_h)+d_h(u_h^k;\tilde{u}_h^{k+1},v_h)=(f,v_h)\quad \forall v_h\in \mathcal {V}_h^0. \end{aligned}$$

In all our calculations we have used $\omega =0.1$, and stopped the iterations when the residual $\varvec{R}^{k}:=(a_h(u_h^{k+1};\psi _i)-(f,\psi _i)_\Omega )_{i=1,\ldots ,\mathrm{dim}(\mathcal {V}_h^0)}$ has an euclidean norm smaller than, or equal to, $10^{-8}$.

**Table 1:** $\varepsilon =10^{-6}$, numerical results for grid (c)

*l*	$\Vert u-u_h\Vert _{0,\Omega }$	Ord.	$|u-u_h|_{1,\Omega }$	Ord.	$\Vert u-u_h\Vert _h$	Ord.
3	0.49391	–	4.38896	–	3.62380	–
4	0.47965	0.04	4.26871	0.04	3.08479	0.23
5	0.19110	1.33	2.71665	0.65	1.08371	1.51
6	0.04080	2.23	1.55469	0.81	0.22671	2.26
7	0.00683	2.58	0.64692	1.27	0.03904	2.54
8	0.00119	2.52	0.27480	1.24	0.00689	2.50

### Convergence for a smooth solution

We take $\varvec{b}=(2,1)$, $\sigma =1$, and different values for $\varepsilon$. We have selected the right-hand-side and boundary conditions in such a way that the solution is given by $u(x,y)=\sin (2\pi x)\sin (2\pi y)$. The meshes used were the three-directional mesh (c) and the non-Delaunay mesh (d) in Fig. 1. In these calculations we have used $\gamma _0^{}=3$ and $p=4$.

The results in Tables 1, 2, 3 and 4 match the theoretical results. In particular we observe a first order convergence in the diffusion-dominated regime for the mesh (c), as predicted by Theorem 4, and a second order convergence in the $L^2$ norm of the error for both the convection and diffusion-dominated regimes. The latter is in accordance with the empirical observations that linearity preservation implies such a convergence. For mesh (d), which is non-symmetric, and hence the method is no longer linearity preserving, we can observe a first order convergence in both regimes. This convergence is not affected by the non-Delaunay character of the mesh.

We finish this example by a deeper study of the behavior of the nonlinear fixed-point iteration with respect to the value of *p*. The results are reported in Table 5. For these results, we have used the three-directional mesh (c), with $l=5$. We can observe that, for the values of *p* ranging from 1 to 10 the iterations needed to reach convergence are essentially independent of the value of *p*. This behavior is kept until a value around 20, and then some non-convergence is observed in the scheme. Here, by non-convergence we mean that the desired residual reduction has not been achieved after 5000 iterations. The same qualitative behavior has been observed for other meshes, and the two other settings presented later. In those cases, non-convergence has been observed starting at values of about 10 or 15, depending on the case. Then, we believe that it is safe to use this scheme for values of *p* not much higher than 10. Of course, further work could be used to find the right damping parameters for each case, but this would come at the price of having to perform much more iterations.

**Table 2:** $\varepsilon =1$, numerical results for grid (c)

*l*	$\Vert u-u_h\Vert _{0,\Omega }$	Ord.	$|u-u_h|_{1,\Omega }$	Ord.	$\Vert u-u_h\Vert _h$	Ord.
3	0.38594	–	3.48242	–	5.44504	–
4	0.16557	1.22	1.90920	0.87	2.26966	1.26
5	0.03268	2.34	0.89029	1.10	0.92785	1.29
6	0.00612	2.42	0.43637	1.03	0.43912	1.08
7	0.00141	2.12	0.21800	1.00	0.21818	1.01
8	0.00035	2.02	0.10903	1.00	0.10904	1.00

**Table 3:** $\varepsilon =10^{-6}$, numerical results for grid (d)

*l*	$\Vert u-u_h\Vert _{0,\Omega }$	Ord.	$|u-u_h|_{1,\Omega }$	Ord.	$\Vert u-u_h\Vert _h$	Ord.
3	0.48754	–	4.33607	–	5.06989	–
4	0.45680	0.09	4.11426	0.08	2.93242	0.79
5	0.17080	1.42	3.15455	0.38	1.05213	1.48
6	0.04330	1.98	2.23948	0.49	0.26065	2.01
7	0.01165	1.89	1.72410	0.38	0.05482	2.25
8	0.00474	1.30	1.63424	0.08	0.02087	1.39

**Table 4:** $\varepsilon =1$, numerical results for grid (d)

*l*	$\Vert u-u_h\Vert _{0,\Omega }$	Ord.	$|u-u_h|_{1,\Omega }$	Ord.	$\Vert u-u_h\Vert _h$	Ord.
3	0.38351	–	3.52996	–	5.57464	–
4	0.16616	1.21	2.00539	0.82	2.41681	1.21
5	0.04513	1.88	0.98086	1.03	1.03172	1.23
6	0.01277	1.82	0.48118	1.03	0.48720	1.08
7	0.00423	1.59	0.23973	1.01	0.24059	1.02
8	0.00163	1.38	0.11982	1.00	0.11998	1.00

**Table 5:** Iterations needed to reach convergence

*p*	1	2	3	4	5	6	7	8	9	10	15	20
Iter.	224	218	261	262	278	286	211	227	197	197	218	206

**Fig. 2:**
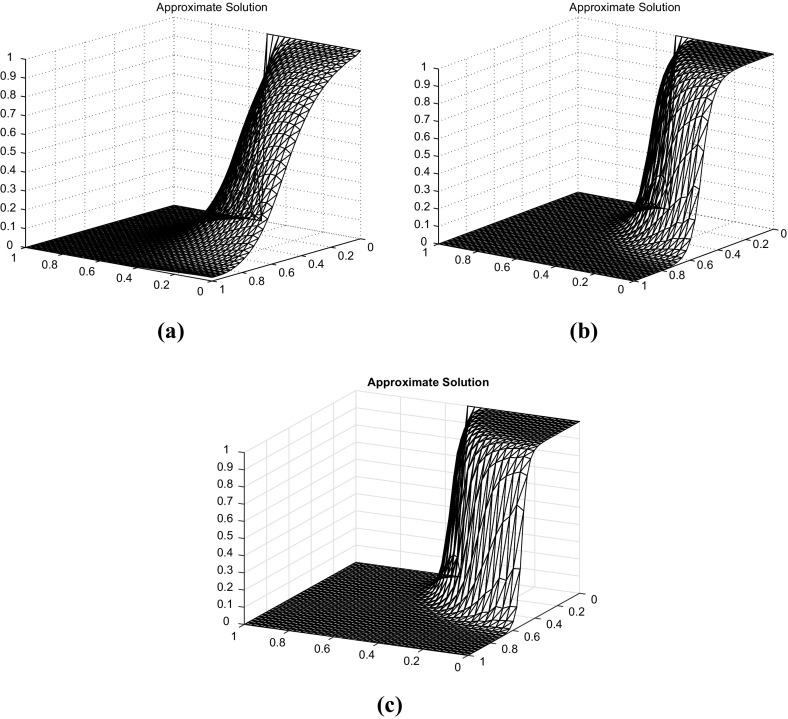
Discrete solution for $p=1$ (*top left*) and $p=4$ (*top right*), and $p=8$ (*bottom*)

**Fig. 3:**
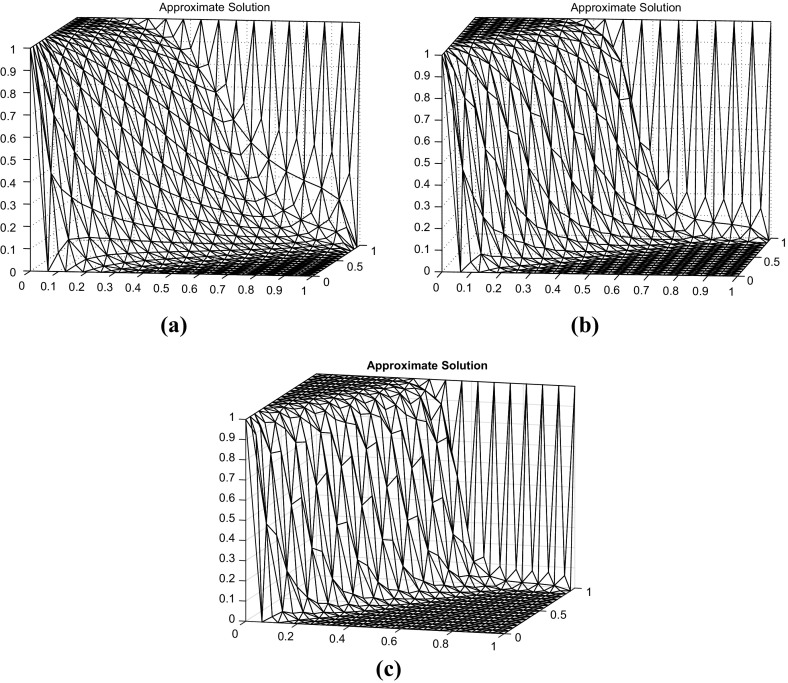
Discrete solution for $p=1$ (*top left*) and $p=4$ (*top right*), and $p=10$ (*bottom*)

### A problem with one inner layer, and a rotating convective field

We use $\varepsilon =10^{-5}$, $f=0$, $\sigma =0$, $\varvec{b}=(-y,x)$, homogeneous Neumann boundary conditions on exit, and

$$\begin{aligned} g(x,y)=\left\{ \begin{array}{ll} 1 &{}\quad \text {if}\; x\le 0.5,\\ 0 &{}\quad \text {else}, \end{array}\right. \end{aligned}$$

as Dirichlet condition at entry. We have solved this problem on a uniform refinement of the three-directional from mesh (c) in Fig. 1. The parameter $\gamma _0^{}$ has been set to 1, and the results show no violation of the DMP. The results for this case are depicted in Fig. 2. We can observe that the increase in the value of *p* provides a solution whose inner layer is much sharper than the choice $p=1$. For both higher values for *p*, a similar behaviour to the one in Table 5 was observed in terms of number of iterations needed for convergence.

### Advection skew to the mesh

We use $\varepsilon =10^{-5}$, $f=0$, $\sigma =0$ $\varvec{b}=\left( \cos \left( \frac{\pi }{3}\right) ,\sin \left( \frac{\pi }{3}\right) \right)$, and

$$\begin{aligned} g(x,y)=\left\{ \begin{array}{ll} 1 &{}\quad \text {if}\; x= 0 \; \text {or}\; y=1,\\ 0 &{}\quad \text {else}, \end{array}\right. \end{aligned}$$

as Dirichlet condition. We have solved this problem on a criss-cross mesh as shown in mesh (a) in Fig. 1. We have used the parameter $\gamma _0^{}=0.75$, and, again, no violations of the DMP have been observed. The results are depicted in Fig. 3, where we can observe much sharper layers (especially the internal one) when higher values for *p* have been used. Again, for both higher values for *p*, a similar behaviour to the one in Table 5 was observed in terms of number of iterations needed for convergence.
